# Definitions and factors associated with subthreshold depressive conditions: a systematic review

**DOI:** 10.1186/1471-244X-12-181

**Published:** 2012-10-30

**Authors:** Mar Rivas Rodríguez, Roberto Nuevo, Somnath Chatterji, José Luis Ayuso-Mateos

**Affiliations:** 1 Instituto de Salud Carlos III, Centro de Investigación Biomédica en Red de Salud Mental, CIBERSAM, Madrid, Spain; 2 Department of Psychiatry, Universidad Autónoma de Madrid, Hospital Universitario de la Princesa, Madrid, Spain; 3 Department of Health Statistics and Informatics, World Health Organization, Avenue Appia 20, Geneva 27, CH 1211, Switzerland; 4 Hospital Universitario de La Princesa, C./Diego de León 62, Madrid, 28006, Spain

## Abstract

**Background:**

Subthreshold depressive disorders (minor and subthrehold depression) have been defined in a wide range of forms, varying on the number of symptoms and duration required. Disability associated with these conditions has also been reported. Our aim was to review the different definitions and to determine factors associated with these conditions in order to clarify the nosological implications of these disorders.

**Methods:**

A Medline search was conducted of the published literature between January 2001 and September 2011. Bibliographies of the retrieved papers were also analysed.

**Results:**

There is a wide heterogeneity in the definition and diagnostic criteria of minor and subthreshold depression. Minor depression was defined according to DSM-IV criteria. Regarding subthreshold depression, also called subclinical depression or subsyndromal symptomatic depression, between 2 and 5 depressive symptoms were required for the diagnosis, and a minimum duration of 2 weeks. Significant impairment associated with subthreshold depressive conditions, as well as comorbidity with other mental disorders, has been described.

**Conclusions:**

Depression as a disorder is better explained as a spectrum rather than as a collection of discrete categories. Minor and subthreshold depression are common conditions and patients falling below the diagnostic threshold experience significant difficulties in functioning and a negative impact on their quality of life. Current diagnostic systems need to reexamine the thresholds for depressive disorders and distinguish them from ordinary feelings of sadness.

## Background

Despite the relevance of Major Depression (or Depressive Episode) as a highly prevalent condition in clinical practice and community settings, its subthreshold forms that do not meet current classificatory thresholds have been less studied. This in large part is due to current diagnostic systems which set the boundary of the disorder based on the presence of a certain number of symptoms. Consequently, persons falling below the threshold are not recognized in primary care settings or community surveys and often not included in biological (imaging and genetic) studies as they are considered to be distinct from those meeting the clinical threshold defined by these systems. Thus, the Diagnostic and Statistical Manual of Mental Disorders, 4^th^ Edition [1] requires the presence of 5 or more symptoms of depression, out of a list of 9, during at least 2 weeks for the diagnosis of Major Depressive Episode, as well as additional criteria such as significant distress and impairment, the absence of direct physiological effects of a substance or a general medical condition that can explain the symptoms. The International Classification of Diseases, 10^th^ edition [2] classifies Depressive Episode into three different groups according to the number of symptoms present, distinguishing between mild, moderate and severe Depressive Episode (with or without psychotic symptoms). Both systems include other diagnostic categories for subjects not meeting the full criteria for the diagnosis of Major Depression or Depressive Episode. The underlying assumption is that categories thus defined have set the threshold at an appropriate place that best separates those with the disorder from those without the disorder in question. These categories then become the basis of both clinical, biological and public health research and the design and delivery of individually directed interventions and policies. In the last two decades, however, increasing recognition of subthreshold forms of depression (minor and subthreshold depression) in various settings has led to attempts to delineate and describe them better and to highlight the necessity of studying their characteristics as a significant clinical entity [3].

There is a wide range of definitions of these subthreshold conditions, and not all of them share the same criteria related to the number of symptoms needed or the impairment present in individual functioning. This heterogeneity leads to a lack of comparability of studies with regard to identification and management of subthreshold depressive disorders. Further agreement in the definition and conceptualisation of sub-threshold depression is also needed in order to achieve a better understanding of the boundaries of depression.

The aim of this paper is to carry out a review of studies examining definitions, prevalence and associated factors (impairment, comorbidity, course and outcome) related to subthreshold depression and minor depression from 2001 to now in order to unravel their implications for their place within the classification of depressive disorders.

## Methods

### Selection of studies

A Medline search of the literature published between January 2001 and September 2011 was conducted. The index terms were “minor depression”, “subclinical depression”, “subsyndromal depression”, “subthreshold depression”, “subthreshold depressive symptoms” and “subclinical depressive conditions” and those terms were searched both in the title and in the abstract. We carried out the same search in PsychInfo and we found a near complete overlap and no papers of significance that met our selection criteria were missed. We included original researches (observational and epidemiological studies) examining definition, prevalence and associated characteristics of minor and subthreshold depression in general population (not in specific age ranges), in both community and primary care settings. Papers written in a language other than English and Spanish were excluded. Bibliography of retrieved papers was examined. 597 papers were considered. 19 studies were selected as they were population-based studies examining subthreshold depressive conditions in general population. The remaining 578 papers were excluded due to several reasons: the type of paper; restriction to specific age ranges, evaluation of interventions (effectiveness of different therapies); studies of the biology of subthreshold depressive disorders; or studies of the psychometric properties of instruments. The data were selected, extracted and analysed by an investigator (MSc in Psychology) and the quality of papers was evaluated in terms of the quality of journals they were published (journals indexed for Medline and peer-reviewed papers) ( Additional file 1: Annexe 1 and Additional file 2: Annexe 2).

## Results

### Nomenclature

Many definitions and names were associated with these conditions (see Table 1). Minor depression was defined according to DSM-IV criteria in the nine studies examining it: at least two weeks of symptoms but the total number of symptoms not exceeding 4 i.e [4-6]. Subthreshold depression (also named “subsyndromal symptomatic depression” in one study, “subsyndromal depression” in four studies, “subclinical depression” in one study and “nonspecific depressive symptoms” in another one) was defined in most cases as depressed mood or loss of interest but having less than five more symptoms or not reporting significant impairment. Despite the heterogeneity of conceptualizations, several studies shared the definition of subsyndromal symptomatic depression proposed by Judd *et al.* in 1994 [7]: “any two or more simultaneous symptoms of depression, present for most or all of the time, at least two weeks in duration, associated with evidence of social dysfunction, occurring in individuals who do not meet criteria for diagnoses of minor depression, major depression, and/or dysthymia” [8,9].

**Table 1:** Definition and duration of minor and subsyndromal depressive disorders

**Name**	**Threshold**	**Symptom set**	**Duration**
Minor depression (Al-Windi, [10])	Total number of symptoms not exceeding 4	DSM-IV	2 weeks
Minor depression (Vuorilehto *et al.*, [11])	Two to four depressive symptoms (at least one core symptom)	DSM-IV	2 weeks
Subsyndromal depression (Vuorilehto *et al.*, [11])	Two to four current depression symptoms (at least one core symptom) and fulfilling the criteria of lifetime MDD	DSM-IV	2 weeks
Subthreshold depression (Rucci *et al.*, [12])	Any of the three symptoms (criterion B) plus three or more symptoms (criterion C)	ICD-10 depressive episode	None
Minor depression (Dubini *et al.*, 2001 [13])	Two to four depressive symptoms (at least one core symptom)	DSM-IV	2 weeks
Subsyndromal symptomatic depression (Forsell, [8])	Two or more symptoms of depression present most or all the time and social impairment due to them	DSM-III major depressive disorder	2 weeks
Nonspecific depressive symptoms (Backenstrass *et al.*, [14])	Two or more symptoms of depression present more than half the days, must not meet the A criterion (depressed mood or anhedonia) but the C criterion (impairment) has to be answered	DSM-IV major depressive disorder	2 weeks
Subsyndromal depression (da Silva Lima *et al.*, [15])	Not fulfilling the CIDI criteria for major depression	CIDI major depression	None
Subthreshold depresión (Fergusson *et al.*, [16])	Depressed mood or loss of interest but having less than five more symptoms or not reporting significant impairment	DSM-IV major depressive disorder	2 weeks
Subsyndromal depresión (Goldney *et al.*, [9])	Two or more depressive symptoms present for all or most of the time but not meeting the criteria for major depression, minor depression or dysthymia	DSM-III-R major depressive disorder	2 weeks
Subsyndromal depression (Ayuso-Mateos *et al.*, [17])	Between one and four depressive symptoms during most of the day and not meeting the criteria for depressive episode or brief episode	ICD-10	2 weeks
Minor depression (Lamers *et al.*, [18])	Two to four depressive symptoms (at least one core symptom)	DSM-IV	2 weeks
Subthreshold depression (Regeer *et al.*, [19])	Not fulfilling the CIDI criteria for Major Depression	DSM-III-R/IV Major Depressive Disorder	2 weeks
Minor depression (Cuijpers *et al.*, [20])	One of the key symtoms plus at least one other symptom but the total number do not exceed 4.	DSM-III-R	2 weeks
Minor depression (De Graaf *et al.*, [4])	2 to 4 symptoms including one key symptom	DSM-IV	2 weeks
Minor depression (Fils *et al.*, [21])	Having reported dysphoric mood or anhedonia with significant life interference and at least 2 but no more than 4 depressive symptoms	DSM-IV	2 weeks
Minor depression (Jackson *et al.*, 2007 [22])	Two to four depressive symptoms (at least one core symptom)	DSM-IV	2 weeks
Minor depressive disorder (Tamburrino *et al.*, 2009 [23])	At least 2 (but less than 5) of the symptoms in Criterion A for a major depressive episode. At least one key symptom.	DSM-IV-TR	2 weeks
Subclinical depression (Gómez- Restrepo *et al.*, [24])	Less symptoms and less severity than depression	ICD-10	-
Subthreshold depression (Baumeister and Morar, [25])	MD criteria fulfilled except for the clinical significance criteria	DSM-IV	2 weeks

Some definitions explicitly excluded depressed mood or anhedonia as an inclusion criterion for subthreshold depressive conditions [14], whereas other studies did include those symptoms [16]. The minimum number of symptoms required for the diagnosis ranged from two to five, the most common minimum being two.

### Prevalence

As regards prevalence rates, they were highly variable across the various studies, ranging in primary care from 1.3 % [11] to 17% [21] for minor depression. In community settings they ranged from 2.8% [18] to 6.1% [20]. Prevalence rates for subthreshold depression ranged from 2.9% [11] to 9.9% [12] in primary care and from 1.4% [24] to 17.2% [19] in community settings (Table 2).

**Table 2:** Prevalences and settings

**Symptom set**	**Sample/population**	**Prevalence**
Minor depression (Al-Windi, [10])	Primary care	12.1%
Subthreshold depression (Rucci *et al.*, [12])	Primary care	9.9%
Minor depression (Dubini *et al.*, 2001 [13])	Community	2.9%
		15.9%
Minor depressive disorder (Tamburrino *et al.*, 2009 [23])	Primary care	
Subsyndromal symptomatic depression (Forsell, [8])	Community	5% (wave 1) 5.5% (wave 2)
Nonspecific depressive symptoms (Backenstrass *et al.*, [14])	Primary care	9.1%
Minor depression (Vuorilehto *et al.*, [11])	Primary care	1.3%
Subsyndromal depression (Vuorilehto *et al.*, [11])	Primary care	2.9%
Minor depression (De Graaf *et al.*, [4])	Community	3%
Subsyndromal depression (Da Silva Lima *et al.*, 2007 [15])	Primary care	6.2%
Subthreshold depression (Fergusson *et al.*, [16])	Community	7.3%
Subsyndromal depression (Goldney *et al.*, [9])	Community	12.9%
Subsyndromal depression (Ayuso-Mateos *et al.*, [17])	Community	2.8%
Minor depression (Cuijpers *et al.*, [20])	Community	6.1%
Subthreshold depression (Regeer *et al.*, [19])	Community	17.2% at baseline
Minor depression (Lamers *et al.*, [18])	Community	2.8%
Minor depression (Fils *et al.*, [21])	Primary care	17%
Subclinical depression (Gómez- Restrepo *et al.*, [24])	Community	1.4%
Minor depression (Jackson *et al.*, 2007 [22])	Primary care	10.4%
Subthreshold depression (Baumeister and Morar, [25])	Community	0.7% clinical significance criteria 1.8% symptom count

### Duration

Regarding subthreshold depression, two studies did not require a minimum duration of symptoms [12,15]. In the eight studies that did report a duration criterion, at least two weeks of symptoms were needed. In the nine studies examining minor depression a minimum duration of two weeks was required.

### Demographics

#### Gender

Minor depression is overrepresented in women in five out of the six studies examinig it, with percentages of 78.9% in Al-Windi [10], 72% in Fils *et al.*[21] and 56.1% in De Graaf *et al.*[4]; and so is subthreshold depression in four studies, such as the 79.2% reported in Da Silva Lima and De Almeida Fleck [15].

#### Family status

Three studies examined this issue in minor depression*:* Fils *et al.*[21] reported that minor depression was more frequent in married people than in divorced/separated/widow (47% vs. 31%), De Graaf *et al.*[4] found a 68.3% of people with minor depression to have a partner and Cuijpers *et al.*[20] also found a higher prevalence in people in this familiar situation.

#### Economic and employment status

Goldney *et al.*[9] found no significant differences in those with subsyndromal depression compared with no depressive patients regarding socioeconomic status. Regarding employment status, two studies examined this issue. Fils *et al.*[21] reported a higher proportion of unemployed respondents (47%) in a sample of people with minor depression. However De Graaf *et al.*[4] found 83.4% of their sample to be employed.

#### Disability and impairment associated

There is an association between quality of life and the presence of depressive symptoms not fulfilling the criteria for the diagnosis of Major Depression or depressive episode. Rucci *et al.*, [12] found that disability in daily activities was increased in individuals with subthreshold depression, which was also associated with significant psychological distress and poor health perception. In 2007, Da Silva Lima and De Almeida Fleck [15] stated that patients with subsyndromal depression had a lower quality of life than subjects without depression while patients with major depression had the lowest quality of life. Goldney *et al.*[9] reported that the average of days in the previous month in which patients with subsyndromal depression were unable to work was higher compared to control subjects. They found a continuum of disability related to depression with those with major depression showing the most severe level impairment. In elderly patients Chachamovich *et al.*[26] found that subthreshold levels of depression were associated with a decreased quality of life and negative attitudes toward aging in a sample of older adults from 20 different countries, along the same line as the previous studies of Beekman *et al.*[27], which also showed that in older adults minor depression was associated with higher levels of disability and well-being. Similarly, Hybels *et al.*[28] found that subthreshold depression in older adults was associated with impairment in physical functioning, disability days and poorer self-rated health.

Regarding minor depression, Cuijpers *et al.*[20] found that functional disability was significantly worse than in those patients with only depressive symptoms but better than in patients with a full episode of major depression along a continuum. Howland *et al.*[29] to the contrary, stated that patients with minor depression had a level of functional impairment comparable to those with major depression. Nieremberg *et al.*[30] suggested that decreased quality of life and psychological well-being may be an intrinsic cognitive aspect of minor depression. As Ayuso-Mateos *et al.*[17] found, subthreshold depressive conditions produce a decrement in health status that does not differ within levels of depression but is significant when compared to non-depressed individuals.

#### Health service use

In 2004, Goldney *et al.*[9] concluded that patients with subsyndromal depression had a significantly greater use of health services than those with no depression. Cuijpers *et al.*[20] also found a higher health service use in patients with minor depression compared to non-depressed people. However, Baumeister and Morar [25] found only a weak association between subthreshold depression and increased health care utilization.

#### Comorbidity

Comorbidity with other physical and mental disorders, is frequently associated with sub-threshold depressive disorders. Rucci *et al.*[12], in their study of the prevalence and associated characteristics of subthreshold psychiatric disorders in primary care, found comorbidity with one or more ICD-10 disorders in 39.6% of individuals with subthreshold depression.

## Discussion

Current classificatory systems need to revisit issues of setting the threshold for depressive disorders both in terms of duration and persistence of symptoms, as well as with regard to the number of symptoms required to qualify for a diagnosis. This is because, as shown in our review, these ‘formes-frustes’ of full blown episodes of depression are common and are associated with significant disability and have significant impact on individual health status. Moreover, by not considering the entire spectrum of depression, classificatory schemes that are used for research may fail to detect biological associations that may underlie the entire spectrum of this condition. Currently, subthreshold depression is defined in heterogeneous ways, a fact which makes it very difficult to discern a clinical category that is useful in day to day practice. In addition to research settings, subthreshold depression is of particular importance in primary care settings since a large majority of patients with this condition are likely to first seek help in primary care and they are likely to form the bulk of persons with depression seeking care [31]. Sensitising primary care providers to these conditions may help in early recognition of depression, delivery of interventions, both pharmacological and interpersonal or problem solving therapies, and perhaps in the identification of persons who are at highest risk of worse outcomes in the future. This would then have the potential of preventing secondary disability associated with depressive disorders. Cuijpers *et al.*[32] have reported the positive effects of psychological treatment for subthreshold depression including its effect in reducing the risk of developing major depression. In addition, the need to better measure the continuum of depression severity has been emphasized while evaluating the effects of antidepressant interventions as current approaches may underestimate the efficacy of antidepressants in less severe forms of depression [33]. However, it is important to distinguish between ordinary human suffering and depressive states, which are qualitatively different, in order to avoid medicalisation of normal adversities and difficulties causing distress and discomfort [34]. Comparing prevalence rates of subthreshold depression based on symptom counts alone with dimensional clinical significance criteria, Baumeister and Morar [25] found lower prevalence rates when clinical significance was considered. In a recent editorial, Maj [35] mentions three possible ways to determine when depression becomes a disorder. The first one emphasises the context where depressive symptoms occur, the second one focuses on the qualitative difference between ordinary sadness and depressive feelings and the last one proposes a distinction based on pragmatic grounds. This last option conceptualises depression as a continuum and two thresholds are proposed: one determining a clinical condition that deserves clinical attention and the other that enables decisions regarding when pharmacological intervention may be needed.

We have only considered the quality of the journal of publication in order to select the papers (journals indexed for Medline and peer-reviewed papers) as we considered it enough quality guarantee. However, we did not examine in depth other measures of individual studies such as power calculation, method of assessment, etc., which means a limitation of the present study.

## Conclusions

The aim of this paper is to consider the question of the boundaries between depression, its subthreshold conditions and normal suffering. To do so we have reviewed literature on this topic and showed data on prevalence, number of symptoms, duration, impact on quality of life and other factors associated. There is a wide heterogeneity of definitions of subthreshold depression considering nomenclature and number of symptoms needed. Nevertheless, all studies report an impact on quality of life and a decrement in health status associated to these conditions. This may lead to support the idea of a continuum of the depressive spectrum and functioning, ranging from non-depression (no impact on quality of life and functioning) to major depression. Categorical models of classification of mental disorders underestimate the importance of subthreshold conditions by not considering their impact on the lives of individuals. Dimensional approaches eliminate an arbitrary threshold and ease the boundaries of mental disorders. This improves precision and mild states of the illness are better identified. Depression seems to be a continuous entity instead of a collection of categories each with its clearly defined boundaries [36,37]. Disability associated with depression is also continuously distributed [20]. Although subthreshold depressive conditions have a smaller impact on quality of life than Major Depression, the impact is, nonetheless, significant as compared to non-depressed subjects [38]. However recent studies show that the impact on health status does not differ significantly between levels of depression [17,39,40] but, rather, between depressed and non-depressed subjects. So, if a boundary has to be established, it should be between depressive disorders and asymptomatic states with the accompanying implications for management.

The distinction with ordinary suffering inherent to several situations is crucial for its implications in diagnosis and treatment. There is a risk of medicalising normal reactions to adversity and it is difficult many times to distinguish this issue. It calls to the knowledge and responsibility of clinicians and therapists to decide whether individual reactions are understandable according to their vital situation or on the contrary they imply a certain degree of psychopathology and are qualitatively different from them.

Proposals to integrate subthreshold depressive conditions in future classificatory systems are being developed. In the DSM-V Development Web site of the American Psychiatric Association [41] there are several proposals to improve the “Depressive disorder not otherwise specified” category in DSM-IV, renaming it as “Depressive conditions not elsewhere classified”.

Future recommendations in the field of subthreshold depressive conditions involve reaching an agreement in the definition of these conditions in terms of their operationalization, number of symptoms and duration required for the diagnosis. Revising the systems of diagnosis and classification of mental disorders is advisable in order to achieve a better understanding of the nature of depression.

## Competing interests

All authors declare that they have no conflicts of interest.

## Authors’ contributions

MRR managed the literature searches and wrote the first draft of the manuscript. JLAM, RN and SC revised the manuscript and contributed to write the second draft. All authors contributed to and have approved the final manuscript.

## Pre-publication history

The pre-publication history for this paper can be accessed here:

http://www.biomedcentral.com/1471-244X/12/181/prepub

## Supplementary Material

Additional file 1 **Annexe 1.** Number of papers excluded and why. Click here for file

Additional file 2 **Annexe 2.** PRISMA 2009 Flow Diagram. Click here for file
