# Supplementary material for: Definitions and factors associated with subthreshold depressive conditions: a systematic review
Source: BMC Psychiatry. 2012 Oct 30;12:181. doi: 10.1186/1471-244X-12-181 (PMC3539957; doi:10.1186/1471-244X-12-181)
Supplement: Additional file 1 — Annexe 1. Number of papers excluded and why. [file 1471-244X-12-181-S1.doc]

Annexe 1: Number of papers excluded and why.

Papers retrieved: 597

Papers selected: 19

Papers excluded: 578

Not written in English or Spanish: 2 (Dutch)

Letter to the editor: 7

Review: 20

Qualitative study: 22

Biological studies: 30

Psychometrical properties of instruments: 40

Concomitant medical illness: 171

Psychological interventions: 19

Pharmacological interventions:44

Other interventions:27

Young population:19

Elder population:117

Middle-aged population:5

Women: 34

Not humans (rats): 1

Neuropsychological functioning: 20

**Search strategy:**

((((minor depression[Title/Abstract]) OR subthreshold depression[Title/Abstract]) OR subclinical depression[Title/Abstract]) OR subsyndromal depression[Title/Abstract]) OR subtreshold depressive conditions[Title/Abstract])))

Limits:

Publication date: From 01/01/2001 to 01/09/2011
