# Supplementary material for: Definitions and factors associated with subthreshold depressive conditions: a systematic review
Source: BMC Psychiatry. 2012 Oct 30;12:181. doi: 10.1186/1471-244X-12-181 (PMC3539957; doi:10.1186/1471-244X-12-181)
Supplement: Additional file 2 — Annexe 2. PRISMA 2009 Flow Diagram. [file 1471-244X-12-181-S2.doc]

**Annexe 2**


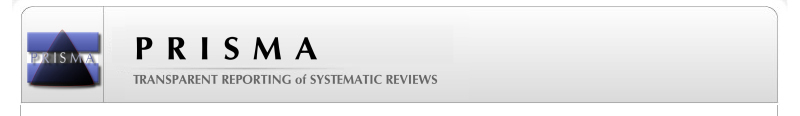
**PRISMA 2009 Flow Diagram**

**Screening**

**Included**

**Eligibility**

**Identification**

Additional records identified through other sources
(n = 0 )

Records after duplicates removed
(n = 597)

Records screened
(n = 597)

Records excluded
(n = 0 )

Full-text articles assessed for eligibility
(n =19 )

Full-text articles excluded, with reasons
(n = 578 )

Studies included in qualitative synthesis
(n =19 )

Studies included in quantitative synthesis (meta-analysis)
(n = 0 )
